# Dysfunctional mitochondrial bioenergetics and the pathogenesis of hepatic disorders

**DOI:** 10.3389/fcell.2015.00040

**Published:** 2015-06-25

**Authors:** Christopher Auger, Azhar Alhasawi, Manuraj Contavadoo, Vasu D. Appanna

**Affiliations:** Faculty of Science and Engineering, Laurentian UniversityGreater Sudbury, ON, Canada

**Keywords:** mitochondrial dysfunction, energy, metabolism, liver disorders, hepatocytes

## Abstract

The liver is involved in a variety of critical biological functions including the homeostasis of glucose, fatty acids, amino acids, and the synthesis of proteins that are secreted in the blood. It is also at the forefront in the detoxification of noxious metabolites that would otherwise upset the functioning of the body. As such, this vital component of the mammalian system is exposed to a notable quantity of toxicants on a regular basis. It therefore comes as no surprise that there are over a hundred disparate hepatic disorders, encompassing such afflictions as fatty liver disease, hepatitis, and liver cancer. Most if not all of liver functions are dependent on energy, an ingredient that is primarily generated by the mitochondrion, the power house of all cells. This organelle is indispensable in providing adenosine triphosphate (ATP), a key effector of most biological processes. Dysfunctional mitochondria lead to a shortage in ATP, the leakage of deleterious reactive oxygen species (ROS), and the excessive storage of fats. Here we examine how incapacitated mitochondrial bioenergetics triggers the pathogenesis of various hepatic diseases. Exposure of liver cells to detrimental environmental hazards such as oxidative stress, metal toxicity, and various xenobiotics results in the inactivation of crucial mitochondrial enzymes and decreased ATP levels. The contribution of the latter to hepatic disorders and potential therapeutic cues to remedy these conditions are elaborated.

## Introduction

As the metabolic hub of the human body, the liver is responsible for the regulation of several biological processes. Roles undertaken by this organ include the neutralization of toxic substances, glycogen storage and hormone production along with fat, glucose and alcohol metabolism (Rui, 2014). It acts as the metabolic gatekeeper between the intestines and blood circulation. It also ensures that toxins are broken down into innocuous compounds to safeguard the organism from harm. For instance, the versatile enzyme cytochrome P450 participates in the metabolic deactivation of thousands of endogenous and exogenous compounds such as bilirubin and drugs, respectively (Chavan et al., 2015). Indeed, exposure to noxious metabolites puts the liver at a greater risk of injury and dysfunction than other internal organs. Hence, it is not surprising that it is an organ capable of natural regeneration (Louvet and Mathurin, 2015). Hepatocytes, the functional units of the liver, make up 70–85% of the mass of this organ and are most susceptible to cellular damage (Mailloux et al., 2011a). Perturbations in the capacity of these cells to execute the biological functions of the liver can give rise to cholestatic and fatty liver disease, diabetes and cancer, among others (Degli Esposti et al., 2012). In order to avoid these afflictions, hepatocytes require a substantial amount of ATP to orchestrate its participation in an extensive amount of biological processes. As such, this universal energy currency is synthesized at a rate of approximately 30 mM/min, with the majority originating from oxidative phosphorylation (Schmid et al., 2008). Defective mitochondria and decreased ATP synthesis are the hallmarks of numerous pathological conditions (Iommarini et al., 2015; Peralta et al., 2015; Torraco et al., 2015).

**Figure 1:**
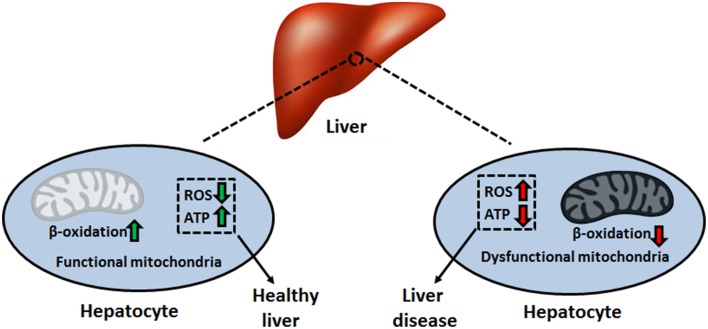
**Mitochondrial dysfunction and disease pathogenesis**. Impaired bioenergetics and increased superoxide leakage stemming from a faulty electron transport chain can initiate and promote the progression of multiple liver disorders. ATP, adenosine triphosphate; ROS, reactive oxygen species.

Mitochondria are vital organelles that harbor the required machinery to perform oxidative phosphorylation. The electron transport chain (ETC), consisting of complexes I–V, couples electron transport to the synthesis of ATP in the mitochondrial matrix (Lemire et al., 2009). This aerobic process requires a reducing component, which comes in the form of the electron carriers NADH and FADH_2_. The generation of these moieties proceeds via the tricarboxylic acid (TCA) cycle, a series of eight enzymatic reactions also residing in the mitochondrion (Lemire and Appanna, 2011). Given the diverse functional roles of the liver, which includes the production of key digestive compounds, cholesterol synthesis, and ammonia removal, hepatocytes contain large amounts of mitochondria to fulfill their bioenergetic demands (Song et al., 2013). In addition, this organelle plays key parts in intrinsic apoptosis, heme synthesis, calcium signaling, and β-oxidation, rendering it indispensable to the modus operandi of the liver (Grattagliano et al., 2011). Hence, mitochondrial disruption tends to provoke and aggravate liver disorders such as insulin resistance, hepatocellular carcinoma (HCC), alcoholic liver disease (ALD), and non-alcoholic fatty liver disease (NAFLD) (Figure 1) (Galloway and Yoon, 2013). In this review, we elaborate on the biomolecular events that orchestrate the pathophysiology of these disorders. These include the uncontrolled generation of reactive oxygen and nitrogen species (ROS and RNS, respectively), inactivation of key transcription factors involved in mitochondrial biogenesis and anaerobiosis as well as signaling cascades which trigger apoptosis and necrosis. At the heart of these biochemical events lies the mitochondrion, the cellular energy machine, whose proper function governs the well-being of the liver.

## Mitochondrial ROS production and defense

Estimates indicate that approximately 0.2–0.5% of the oxygen consumed by the mitochondrion is converted into ROS, a number that is tissue-dependent and varies with the redox state of the organelle (Chance et al., 1979). Heavy metals, ethanol, and oxidative stress are known to exacerbate ROS formation (Auger et al., 2013; Lemire et al., 2014). ROS leakage in the ETC occurs predominantly at the sites of complex I (NADH ubiquinone oxidoreductase) and complex III (ubiquinone cytochrome c oxidoreductase) in the form of superoxide (O^•−^_2_) (Miwa and Brand, 2005; Treberg et al., 2011). However, auxiliary sites of ROS production, such as pyruvate dehydrogenase (PDH), alpha-ketoglutarate dehydrogenase (KGDH), and succinate dehydrogenase (SDH), do add to the oxidative burden of this organelle (Murphy, 2012). Within the mitochondrial matrix, Mn-dependent superoxide dismutases (MnSOD) are known to catalyze the dismutation of O^•−^_2_ to hydrogen peroxide (H_2_O_2_) that can be readily detoxified by glutathione peroxidase (GPX) (Wallace et al., 2010). However, if the concentration of H_2_O_2_ is not controlled, it diffuses to the cytoplasm and participates in a series of reactions generating other reactive compounds, such as hydroxyl radical (^•^HO). The indiscriminate nature of ^•^HO, which reacts with lipids, nucleic acids, and amino acids, renders it short lived but highly dangerous in biological systems (Imlay, 2013). Furthermore, the diffusion-limited reaction of O^•−^_2_ with the gaseous signaling compound nitric oxide (NO^•^) forms peroxynitrite (ONOO^−^) (Auger and Appanna, 2015). The generation of this oxidant and nitrating agent can have potentially toxic ramifications via the nitration of tyrosine residues and s-nitrosylation of cysteine moieties on vital proteins in the mitochondrion (Pacher et al., 2007). Moreover, exposure to bioavailable cationic metals such as Al and Zn can lead to an increase in ROS and displace Fe from the active site of some proteins, such as aconitase (ACN) (Lemire et al., 2008; Han et al., 2013). Free Fe poses a threat to cells due to its participation in Fenton chemistry, which further increases the concentration of detrimental ^•^HO (Ahlqvist et al., 2015).

**Figure 2:**
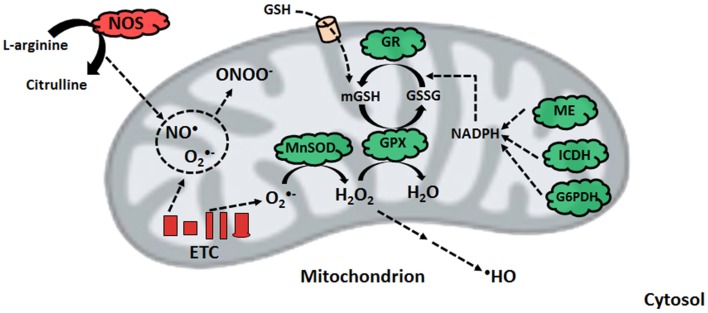
**Nitro-oxidative stress production and detoxification**. Within the mitochondrion, superoxide from electron transport chain activity can be readily converted to peroxide or react with nitric oxide to form peroxynitrite. To limit peroxide diffusion and synthesis of the hydroxyl radical, organisms maintain a pool of mitochondrial glutathione, whose renewal is orchestrated by glutathione reductase with the help of various NADPH-generating enzymes. ETC, electron transport chain; G6PDH, glucose-6-phosphate dehydrogenase; GPX, glutathione peroxidase; GR, glutathione reductase; GSH, reduced glutathione; GSSG, oxidized glutathione; ICDH, NADP-dependent isocitrate dehydrogenase; ME, malic enzyme; MnSOD, manganese superoxide dismutase; NADPH, reduced nicotinamide adenine dinucleotide phosphate; NOS, nitric oxide synthase.

Nitro-oxidative stress occurs when the production of these radicals is in excess of the ability of the cell to limit their reactivity. Given that the majority of cellular ROS is generated in the mitochondrion, which houses high-priority targets of these moieties, this organelle requires an intricately-regulated antioxidant system (Song et al., 2013). The aforementioned MnSOD, in tandem with mitochondrial glutathione (mGSH) as well as glutaredoxin and thioredoxin systems are responsible for limiting the accumulation of O^•−^_2_ and H_2_O_2_. GSH, which is synthesized in the cytosol and imported into the mitochondrion, reduces peroxide concentrations with the assistance of GPX, or peroxiredoxin, subsequently producing oxidized glutathione (GSSG) (Mari et al., 2009). The latter is converted back into GSH via NADPH-dependent glutathione reductase, thus maintaining the GSH redox cycle. The pool of mGSH represents 10–15% of the total cellular GSH content (Ribas et al., 2014). GSH renewal is assured by a number of NADPH-generating enzymes in the mitochondrion, including malic enzyme (ME), NADP-dependent isocitrate dehydrogenase (ICDH), and a matrix-localized glucose-6-phosphate dehydrogenase (G6PDH) (Figure 2) (Mailloux and Harper, 2010; Yin et al., 2012a). In humans, the absence of various metabolic strategies aimed at neutralizing oxidative stress that are operative in other organisms gives rise to ineffective mitochondrial enzymes and disease can ensue (Chenier et al., 2008; Mailloux et al., 2011b).

## Mitochondrial ROS and RNS targets

When the production of radical compounds outmatches the ability of antioxidants to scavenge them, a number of biomolecules within the mitochondrion are at risk. Superoxide, the organelle's inherent ROS species, reacts primarily with iron–sulfur (Fe–S) clusters in the absence of NO^•^. Fe–S cluster-containing proteins in mitochondria include ACN, SDH, fumarase (FUM), and complex I (James et al., 2012). Interference with the catalytic ability of these proteins affects the flux of carbon through the TCA cycle, thus diminishing NADH production and subsequently, ATP synthesis (Mailloux et al., 2006). Other enzymes that are amenable to redox reactions include the lipoic acid-containing subunits (E2) of KGDH and PDH (Ambrus et al., 2009; Auger et al., 2011). Under pathological conditions, ROS and RNS inhibit the activities of MnSOD, glutathione reductase and glutathione peroxidase, further aggravating the noxious effects of these radicals (Song et al., 2013). Lipid peroxidation, a signature reaction of hydroxyl radical, produces cytotoxic lipid peroxides such as malondialdehyde (MDA). The latter can form covalent protein adducts and inactivate key enzymes such as ACN, ATP synthase (complex V), and aldehyde dehydrogenase (ALDH2) (Doorn et al., 2006).

Nitric oxide, whose conventional physiological role is to regulate blood flow and hepatic circulation, can react with heme-containing proteins such as cytochrome c oxidase of the ETC. In response to oxidative and inflammatory stresses, inducible nitric oxide synthase is expressed, leading to a spike in NO^•^ levels, an event linked to the s-nitrosylation of complexes I and IV, as well as creatine kinase (Gross et al., 1996; Auger and Appanna, 2015). Furthermore, ONOO^−^ formation stemming from increased NO^•^ and O^•−^_2_ synthesis can have adverse downstream effects. The reaction of NADH and ONOO^−^ generates NAD^+^ and O^•−^_2_, thus forming a positive-feedback cycle resulting in an oxidative environment (Kirsch and De Groot, 1999). Peroxynitrite also participates in the s-nitrosylation of catalytic cysteine residues on NADP^+^-dependent ICDH and thiol oxidation of the adenine nucleotide translocator (Vieira et al., 2001; Yang et al., 2002). Oxidative modifications of mitochondrial DNA (mtDNA) can further decrease the production of ATP by the ETC. Proteins encoded by mtDNA include 13 polypeptides, all of which are subunits of the oxidative phosphorylation complexes. As mtDNA lacks protective histones and has a lower rate of DNA repair activity compared to the nucleus, deletions and modifications within the mitochondrial genome result in an accumulation of mutations in proteins of the ETC, thus increasing ROS production and diminishing energy levels (Begriche et al., 2011). While it is estimated that each mitochondrion contains 5–10 copies of mtDNA, a number aimed at limiting the mutagenic properties of ROS and other toxicants, adverse effects become pronounced when these copies fall below 20–40% of basal levels (Bohr and Anson, 1999). Deletions in mtDNA lead to the development of neuromuscular disorders such as Kearns-Sayre and Pearson syndrome. One in 4000 children develop mitochondrial disease by the age of 10. Moreover, mtDNA mutations may play a role in the pathogenesis of chronic liver disease (Viscomi et al., 2015) (Table 1).

**Table 1:** **Examples of mitochondrial DNA mutations and their respective liver disorders**.

**Genetic mutation**	**Mitochondrial defect**	Liver dysfunction^*^
A193T	Cytochrome b	Alcoholic liver disease (Wong et al., 2004)
G263A C insertion between 311 and 312 T489C	D-loop	Hepatocellular carcinoma (Nishikawa et al., 2001)
A3243G	tRNA (LeuUUR)	Type II diabetes mellitus (Kim et al., 2008)
G3842A	NADH dehydrogenase (subunit 1)	Hepatocellular carcinoma (Yin et al., 2010)
T6787C	Cytochrome c oxidase (subunit 1)	Hepatocellular carcinoma (Yin et al., 2010)

* *Disease manifested by patient at time of study. Other hepatic disorders resulting from mitochondrial dysfunction may have occurred*.

## Liver mitochondria and diabetes pathogenesis

Diabetes mellitus is a multifactorial metabolic disorder characterized by insensitivity to insulin, a hormone regulating the absorption of glucose from blood to bodily tissues. The liver is a primary target of insulin and glucagon signaling, regulating glycogen synthesis, and gluconeogenesis in hepatocytes. It is a well-recognized fact that increased ROS and diminished mitochondrial ATP production are molecular hallmarks of type 2 diabetes mellitus (T2DM) (Szendroedi et al., 2012). Indeed, diabetic patients have higher levels of mitochondrial protein and lipid oxidation. An increase in mutations and low concentrations of mtDNA have also been reported. More specifically, an augmentation in the lipid peroxidation product 4-hydroxynonenal (4-HN) and DNA oxidation biomarker 8-hydroxy-2′-deoxyguanosine in addition to an up-regulated NADPH oxidase and decreased GSH:GSSG ratio are common symptoms of the disorder (Noriega-Cisneros et al., 2013). Lowered activity of complex I/IV as well as MnSOD are signs of T2DM progression, and likely the result of increased nitro-oxidative stress affecting key moieties such as Fe-S clusters, heme groups, and mtDNA coding for ETC components (Pagano et al., 2014). What remains unclear is whether these biomolecular events precede, accompany or are a consequence of the pathogenesis of diabetes. In Otsuka Long-Evans Tokushima fatty rats, a commonly studied model of obesity and T2DM, hepatic mitochondrial dysfunction occurs weeks in advance of the development of insulin resistance. Lower SOD and increased concentrations of GSSG were detectable at 5 weeks of age. Decreased activity of carnitine palmitoyltransferase I, the rate-limiting step of mitochondrial fatty acid entry, was accompanied by impaired fatty acid oxidation (Rector et al., 2010). Excess energy intake in the form of over-nutrition leads to an increase in circulating free fatty acids. As lipid infusion has been shown to reduce insulin sensitivity, defective lipid metabolism stemming from mitochondrial disorder appears to be a key initiator of insulin resistance and subsequently, T2DM. In the isolated liver, gluconeogenesis, the generation of glucose, is dependent on mitochondrial fat oxidation. It is noteworthy that while β-oxidation and ketogenesis are dysfunctional in the diabetic liver, pyruvate carboxylase, and TCA cycle flux appear to be elevated. This is in contrast to the mitochondria from skeletal muscle of diabetic rat models, where TCA cycle activity appears to be down-regulated. As such, the build-up of NADH and fatty acid concentrations in conjunction with the lowered activity of ETC complexes may further compound the ROS burden, thus bringing about insulin-resistance in the liver of mammalian organisms (Kim et al., 2008). Hence, mitochondrial dysfunction coupled with diminished ATP synthesis is an important contributor to diabetes.

## Obesity and NAFLD

With approximately 15–20% of the Western population affected, NAFLD has become the most common form of chronic liver disease. This underscores the fact that the number of children affected by fatty liver has grown exponentially over the course of the last couple of decades (Wieckowska and Feldstein, 2005; Nassir and Ibdah, 2014b). NAFLD is diagnosed when hepatic fat content exceeds 5% of the liver's total weight in the absence of significant alcoholic intake, viral, and autoimmune components (Nassir and Ibdah, 2014b). The widely accepted hypothesis invoked for the development of NAFLD is the two hits model, whereas the first hit is a metabolic disorder such as insulin resistance and hyperlipidemia. The second hit involves environmental or genetic factors which trigger mitochondrial dysfunction (Tilg and Moschen, 2010). Analogous to the pathogenesis of T2DM, the initial biological events leading to NAFLD are an increase in mitochondrial ROS, defective ATP generation, and deficiencies in β-oxidation (Mantena et al., 2008). In NAFLD models, accumulation of lipid peroxidation and inactivation of key enzymatic entities in the mitochondrion leads to ROS-induced oxidative damage to cardiolipin, a vital phospholipid in the inner mitochondrial membrane. Cardiolipin is necessary for the functioning of ETC complexes as it plays a role in the folding and structures of these moieties (Paradies et al., 2002). As these defects begin to worsen, mitochondrial morphology, and concentration is altered drastically. Electron microscopy in mouse models reveals lowered levels of mitochondria which are swollen and contain paracrystalline inclusions (Wei et al., 2008). Tumor necrosis factor-α (TNF-α), a common cytokine involved in systemic inflammation, plays a role in perturbing mitochondrial morphology and function. TNF-α is elevated in the bloodstream of patients with NAFLD and liver tissue of obese mice, where it is known to induce mitochondrial swelling and perturbations (Hui et al., 2004). The latter leads to defects in ETC activity, either directly or via mtDNA damage, thus decreasing ATP production by liver mitochondria, a phenomenon accompanied by increased oxidative burden (Sanchez-Alcazar et al., 2000). Furthermore, TNF-α has been shown to induce hypoxia-inducible factor-1α (HIF-1α), thus promoting a hypoxic environment and lowered oxidative energy synthesis (Begriche et al., 2006).

**Figure 3:**
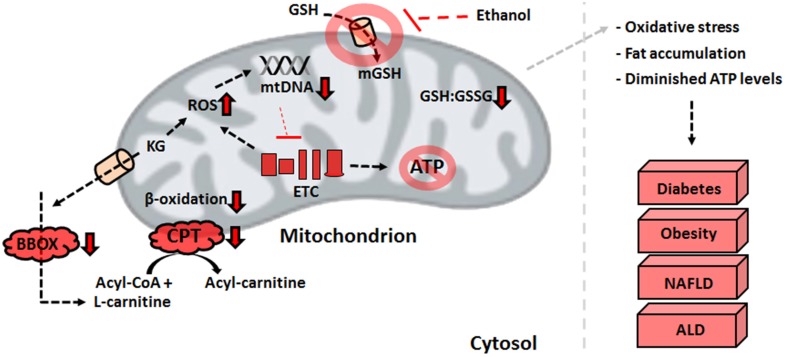
**Defective mitochondrial processes underlying liver disorders**. An increase in ROS resulting from the electron transport chain defects or environmental factors can have detrimental effects on ATP production and β-oxidation, biomolecular events that trigger the development of liver diseases. ALD, alcoholic liver disease; ATP, adenosine triphosphate; BBOX, gamma-butyrobetaine dioxygenase; CPT, carnitine palmitoyltransferase I; ETC, electron transport chain; GSH, reduced glutathione; GSSG, oxidized glutathione; KG, alpha-ketoglutarate; NAFLD, non-alcoholic fatty liver disease; ROS, reactive oxygen species.

The detrimental effects of ROS and RNS within the mitochondria trigger various metabolic changes that limit the production of reducing factors and ATP in the liver. Defective MnSOD and a diminished GSH:GSSG ratio initiate the adoption of novel strategies to limit the accumulation of ROS (Thomas et al., 2015). The down-regulated activities of PDH and KGDH brought about by oxidation of the E2 subunit lead to an accumulation of alpha-ketoglutarate (KG) and pyruvate. This pool of keto acids serves as an antioxidant *in situ*, reacting non-enzymatically with H_2_O_2_ to produce the organic acids succinate and acetate, respectively (Mailloux et al., 2011a; Lemire et al., 2014). While this stratagem alleviates the oxidative burden in hepatocytes, it also has wide-ranging implications for fat oxidation. L-carnitine, an essential metabolite which shuttles fatty acyl compounds to the mitochondrial matrix for further breakdown, is formed via four enzymatic steps in biological systems. The final reaction of carnitine biosynthesis, catalyzed by gamma-butyrobetaine dioxygenase (BBOX), is reliant on KG. As the latter is employed to combat oxidative stress, the formation of carnitine is diminished, which leads to an accumulation of fatty acids. Indeed, the activity of BBOX in hepatocytes is lowered by exposure to the stress of H_2_O_2_ and metal toxicity (Figure 3) (Lemire et al., 2011). Supplementation of KG to injured cells restores the ability to oxidize palmitic acid, further demonstrating the contribution of nitro-oxidative stress to the pathogenesis of obesity and NAFLD (Lemire et al., 2011). The elevated amounts of succinate in the cytoplasm induced by the KG-propelled detoxification of ROS results in the stabilization of hypoxia inducible factor 1-alpha. This promotes anaerobiosis, enhanced glycolysis, and fat accumulation (Mailloux et al., 2009).

As the effects of lowered aerobic metabolism and fat oxidation in addition to increased oxidative stress begin to compound, cellular apoptosis become activated to eliminate damaged cells. In liver cells, mitochondrial dysfunction triggers the intrinsic apoptotic pathway, releasing cytochrome c into the cytosol. Within the mitochondria, the interplay between the anti-apoptotic proteins Bcl-2 and Bcl-xL and the pro-apoptotic proteins Bax and Bak dictate the initiation of cell death (Cory and Adams, 2002). Diminished ATP lowers mitochondrial ability to maintain its structure and function, leading to the activation of mitochondrial permeability transition pore. Inability to prevent cell death in NAFLD ultimately culminates into advanced fibrosis and cirrhosis of the liver, underscoring the significance of alleviating mitochondrial dysfunction to slow the pathogenesis of this disorder (Paradies et al., 2014).

## Alcoholic liver disease

According to the World Health Organization, acute and excessive consumption of alcohol contributes to approximately 5% of global disease and injury. Chronic alcohol drinkers are at risk of developing ALD, steatohepatitis and eventually, cirrhosis of the liver. The latter predisposes the patient to the development of HCC (Rehm et al., 2009). Once in the liver, ethanol is metabolized via alcohol dehydrogenase (ADH), H_2_O_2_-dependent catalase and cytochrome P450 isoforms. The oxidation of ethanol forms the highly reactive acetaldehyde, which is capable of forming protein and DNA adducts, further contributing to tissue damage. While normally, acetaldehyde is rapidly detoxified by mitochondrial ALDH, genetic variations in ALDH, and ADH function as well as increased oxidative stress could inhibit the ability to of the liver to control concentrations of this moiety (Zakhari, 2006). Additionally, it has been proposed that acetaldehyde catabolism increases the pool of mitochondrial NADH, a pro-oxidant which fuels an ineffective ETC and subsequently increases O^•−^_2_ levels (Yu et al., 2010). ALD-affected individuals have enlarged mitochondria, lowered oxidative phosphorylation, and defective fatty acid oxidation. Furthermore, increases in ROS and RNS contribute to deletions and mutations in mtDNA, common symptoms of the prevalent liver disorders (Nassir and Ibdah, 2014a).

Protein deacetylation, which regulates DNA transcription as well as enzymatic activity, is controlled by the sirtuin (SIRT) family. Peroxisome proliferator-activated receptor gamma coactivator 1-alpha (PGC-1α), which is activated by deacetylation, is involved in the stimulation of mitochondrial biogenesis. In rodent models, exposure to ethanol leads to a decrease in SIRT1, which regulates lipid metabolism though sterol regulatory element-binding protein-1c as well as mitochondria biosynthesis through PGC-1α (Fernandez-Marcos and Auwerx, 2011). Moreover, ethanol exposure seems to induce the expression of miR-217, a non-coding micro RNA which further lowers SIRT1 expression and up-regulates the activity of the lipogenic enzymes acetyl-CoA carboxylase and fatty acid synthase while lowering β-oxidation by acyl-CoA oxidase (Yin et al., 2012b).

Hepatocytes from ALD subjects are more susceptible to ROS and RNS toxicity as well as apoptotic or necrotic cell death, due to shortages of ATP and increased lipid peroxidation. To limit such an outcome, the maintenance of mitochondrial redox state by mGSH is paramount. It is widely accepted that transport of GSH from the cytosol to the mitochondrion proceeds via the 2-oxoglutarate carrier, which catalyzes the electroneutral exchange of KG for malate, or other dicarboxylic acids (Coll et al., 2003). Changes in mitochondrial membrane fluidity, such as fluctuations in the cholesterol:phospholipid ratio, greatly impact the ability of membrane proteins, including those involved in substrate transport. In alcohol-fed rat livers, the initial rate of 2-oxoglutarate transport is diminished, negatively affecting the ability of the mitochondrion to import cytosolic glutathione. Indeed, increasing the cytosolic concentrations of N-acetylcysteine, a GSH precursor, does not increase the pool of mGSH in ALD models. However, preventing the loss of membrane fluidity with s-adenosylmethionine restores the ability to transport GSH to the organelle (Mari et al., 2009). Hence, bolstering the antioxidant capability of the mitochondria may limit the cytotoxicity of alcohol, enhance ATP production, and slow the progression to cirrhosis.

## Hepatocellular carcinoma and ATP homeostasis

Accounting for approximately three-fourths of primary liver cancers, HCC is the second leading cause of cancer-related mortality globally. While many cancers have declined due to early treatment and preventative measures, the incidence of HCC is still on the rise, having doubled over the past two decades. Risk factors for HCC include alcohol abuse, NAFLD and hepatitis B and C infections (Forner et al., 2012). Rapidly dividing tumor cells are known to utilize glycolysis as a primary energy source in lieu of oxidative phosphorylation, even in the presence of oxygen (Warburg et al., 1927). This phenomenon, known as the Warburg effect, generally involves mutations in phosphatase and tensin homolog and Ras that activate phosphoinositide 3-kinase to increase glucose uptake. Furthermore, oncogenes such as Myc, which increases glycolytic lactate dehydrogenase, amplify glucose consumption in an aerobic environment (Brandon et al., 2006). To promote this lifestyle, dysfunctional mitochondria are a prerequisite. Indeed, several studies have implicated mitochondrial disorders in the initiation and progression of HCC (Warburg, 1956; Lee and Wei, 2009).

There is a large body of literature linking free radicals and defective antioxidant enzymes to cancer. More specifically, SOD over-expression can inhibit malignant transformation. The reduction of MnSOD activity over the life span of a mouse model correlates to a higher incidence of cancer. Mitochondria-targeted carcinogens, such as arsenic, promote ROS production, a biological event conducive to mtDNA mutations and deletions. In line with what is seen in other cancers, 52% of HCC patients carry at least one point mutation in tumor tissue mtDNA (Yin et al., 2010). These genomic alterations in conjunction with a low mtDNA copy number promote mitochondrial dysfunction via decreased expression and activity of cytochrome oxidase and increased electron leakage from the ETC (Nishikawa et al., 2001). In human hepatoma HepG2 cells, inhibitors of respiration or mtDNA replication are known to induce mitochondrial dysfunction and resistance to the chemotherapy drug cisplatin (Chang et al., 2009). Moreover, certain pathogenic mtDNA mutations, such as the T8993G transversion, promote tumor growth by preventing apoptosis (Shidara et al., 2005). As such, ROS and RNS-mediated mtDNA modifications appear to play a key role in disabling this organelle and facilitating the transition to glycolytic energy-production.

**Figure 4:**
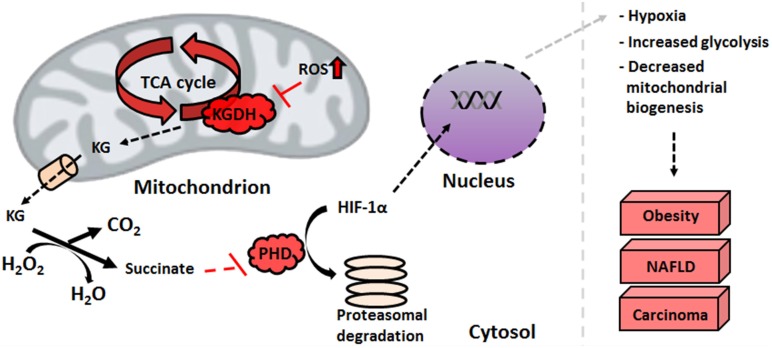
**HIF-1 α stabilization is facilitated by elevated succinate levels**. An increase in mitochondrial nitro-oxidative stress impedes alpha-ketoglutarate dehydrogenase functionality, which leads to the pooling of alpha-ketoglutarate. The latter scavenges peroxide with the subsequent formation of succinate, an inhibitor of HIF prolyl hydroxylases. This stabilizes HIF-1α, protecting it from proteasomal degradation and allowing it to promote hypoxic conditions in the nucleus. HIF-1α, hypoxia-inducible factor; KG, alpha-ketoglutarate; NAFLD, non-alcoholic fatty liver disease; PHD, prolyl hydroxylase; ROS, reactive oxygen species.

In addition to ETC defects, increased oxidative stress wreaks havoc on the TCA cycle enzymes. As described above, KGDH inhibition leads to a pooling of KG in the HepG2 cell line. Non-enzymatic decarboxylation of KG mediated by H_2_O_2_ causes an increase in succinate concentrations (Mailloux et al., 2009). The decreased activity of SDH observed in several types of cancer further contributes to the accumulation of this TCA cycle intermediate (Kim and Dang, 2006). Under normoxic conditions, HIF-1α is rapidly degraded by prolyl hydroxylases (PHDs). By hydroxylating proline residues in the oxygen-dependent degradation domain, PHDs induce the proteosomal breakdown of this oxygen sensor. Succinic acid interferes with the activity of PHDs by perturbing substrate-binding sites within the enzyme, a molecular event which favors HIF-1α stability and subsequently promotes hypoxia via the nuclear localization of HIF-1α (Figure 4) (Mailloux and Appanna, 2007). While in other liver disorders, the dearth of ATP, defective β-oxidation and increased oxidative burden triggers apoptotic pathways and cell death, over-activation of anti-apoptotic mediators such as BcL-X(L), Mcl-1, c-IAP1, and XIAP seen in HCC ensure cell survival and encourages tumor growth (Hoenerhoff et al., 2011).

## Therapeutic cues

Despite the variance in phenotype stemming from the plethora of liver disorders, there exists a significant overlap in the biomolecular pathogenesis at the mitochondrial level. Defective oxidative phosphorylation, increased ROS and RNS, diminished β-oxidation, and enhanced lipogenesis are some of the recurring commonalities across the spectrum of hepatic diseases. Therefore, mitigating the oxidative burden and bolstering the mitochondrion's capacity to produce ATP and break down fatty acids can have significant health benefits. The interplay between mitochondrial function and mitochondrial biogenesis suggests that increasing the number of this organelle can alleviate the symptoms of mitochondrial disorders. Indeed, treatment with thiazolidinediones, synthetic peroxisome proliferator-activated receptor gamma (PPAR-γ) ligands, has been reported to improve insulin resistance partially through increased mitochondrial biogenesis (Bogacka et al., 2005). The antidiabetic drug metformin has also been reported to increase mitochondrial numbers through activation of PGC-1α and adenosine monophosphate kinase (AMPK) signaling, while also lowering ROS concentrations (Kim et al., 2008).

Mitochondria-targeted antioxidants can also be exploited for the remediation of liver disorders. For instance, the beneficial effects of vitamin E and C supplementation on tempering ROS levels are well described (Nassir and Ibdah, 2014b). The ONOO^−^ scavenger metalloporphyrin has positive effects on mitochondrial redox state, as demonstrated by restored activity of ALDH2 and ATP synthase (Moon et al., 2008). Treatment with resveratrol, a polyphenolic antioxidant found in grapes, can prevent high fat diet-induced steatosis through regulation of metabolic regulators of energy such as SIRT1 and AMPK (Poulsen et al., 2012). Keto acid supplementation in the form of KG or pyruvate can relieve nitro-oxidative stress directly via their antioxidant properties while restoring other mitochondrial functionalities indirectly. For example, KG treatment in HepG2 cells increases levels of L-carnitine and restores mitochondrial β-oxidation (Lemire et al., 2011). Pyruvate supplementation drives the pyruvate carboxylase and ME reactions, thus augmenting levels of NADPH and mitochondrial citrate. The latter effluxes to the cytosol and suppresses phosphofructokinase activity, diverting glucose to the production of NADPH via the pentose phosphate pathway. The end result of this metabolic reconfiguration is an increased GSH:GSSG ratio, a parameter leading to reduced oxidative damage. Indeed, pyruvate supplementation suppresses mtDNA damage and protein degradation (Tanaka et al., 2007; Liu et al., 2011).

While supplements may slow the progression of liver disease, preventative measures such as reduced caloric intake, exercise, and lower alcohol consumption are key factors in restoring mitochondrial ability. Exercise and physical activity has been shown to improve mitochondrial function and insulin sensitivity in T2DM patients and subjects with NAFLD. Aerobic exercise may stimulate mitochondrial biogenesis through increased expression of PGC-1α (Kim et al., 2008). Furthermore, endurance exercise and caloric restriction increase mitochondrial size, number, and oxidative phosphorylation, thus improving cellular bioenergetics. Additionally, dietary choices such as increased consumption of polyunsaturated fatty acids and antioxidant-containing foods can prevent protein oxidation and mitochondrial dysfunction (Nassir and Ibdah, 2014b). These lifestyle decisions ultimately lead to higher oxidative ATP production, increased fatty acid oxidation and lower hepatic fat accumulation, thus limiting the pathogenesis of liver disorders.

## Conclusion

**Figure 5:**
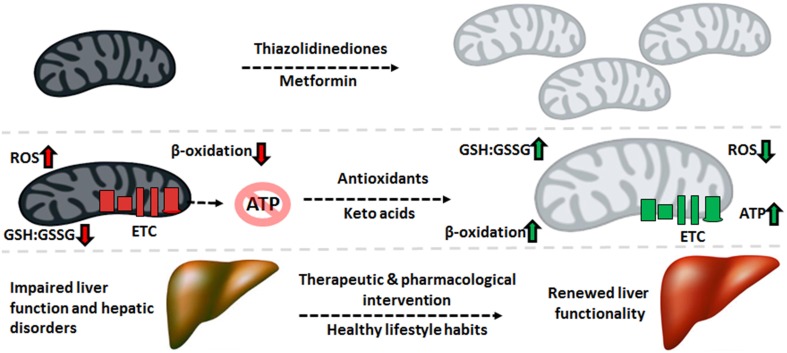
**Therapeutic cues aimed at mitochondrial restoration**. Pharmaceutical compounds which increase mitochondrial biogenesis and mitochondria-targeted antioxidants geared to diminishing the nitro-oxidative burden can be applied to reverse the molecular events underlying the pathogenesis of liver disorders, renewing the function of this crucial organ. ATP, adenosine triphosphate; ETC, electron transport chain; GSH, reduced glutathione; GSSG, oxidized glutathione; ROS, reactive oxygen species.

The pathogenesis of liver disease is a complex and multifactorial process where environmental and genetic signals converge upon the organ, causing failure to accomplish its vast biological functions. While there exists several disparities in the means by which liver disorders progress and present themselves, mitochondrial dysfunction is a common link in most hepatic diseases. The role of this organelle in the aerobic synthesis of ATP, oxidation of fatty acids and apoptotic signaling renders it indispensable to cellular function, particularly in energy-intensive organs like the liver. As such, injury to mitochondria which give way to dysfunction generally precedes the development of such disorders as T2DM, NAFLD, ALD, and HCC. Increased nitro-oxidative stress results in lipid peroxidation and protein oxidation and consequently, inactivation of ETC complexes and mtDNA mutations or deletions. Therapeutic and pharmacological strategies to decrease the oxidative burden and maintain a healthy mitochondrial redox state can halt or reverse the progression of various liver diseases, particularly when combined with healthy lifestyle habits (Figure 5). As such, mitochondria-targeted treatments which bolster the ability of this organelle to execute its functions can be implemented to alleviate liver diseases and thus should be explored more rigorously for their therapeutic potential.

### Conflict of interest statement

The authors declare that the research was conducted in the absence of any commercial or financial relationships that could be construed as a potential conflict of interest.
